# A scoping review assessing the usability of digital health technologies targeting people with multiple sclerosis

**DOI:** 10.1038/s41746-024-01162-0

**Published:** 2024-06-25

**Authors:** Fiona Tea, Adam M. R. Groh, Colleen Lacey, Afolasade Fakolade

**Affiliations:** 1 Department of Neuroscience, Université de Montréal, Montreal, QC Canada; 2 Department of Neurology and Neurosurgery, Montreal Neurological Institute-Hospital, McGill University, Montreal, QC Canada; 3 Department of Psychology, University of Victoria, Victoria, BC Canada; 4 School of Rehabilitation Therapy, Queen’s University, Kingston, ON Canada

**Keywords:** Translational research, Neuroimmunology, Therapeutics

## Abstract

Digital health technologies (DHTs) have become progressively more integrated into the healthcare of people with multiple sclerosis (MS). To ensure that DHTs meet end-users’ needs, it is essential to assess their usability. The objective of this study was to determine how DHTs targeting people with MS incorporate usability characteristics into their design and/or evaluation. We conducted a scoping review of DHT studies in MS published from 2010 to the present using PubMed, Web of Science, OVID Medline, CINAHL, Embase, and medRxiv. Covidence was used to facilitate the review. We included articles that focused on people with MS and/or their caregivers, studied DHTs (including mhealth, telehealth, and wearables), and employed quantitative, qualitative, or mixed methods designs. Thirty-two studies that assessed usability were included, which represents a minority of studies (26%) that assessed DHTs in MS. The most common DHT was mobile applications (*n* = 23, 70%). Overall, studies were highly heterogeneous with respect to what usability principles were considered and how usability was assessed. These findings suggest that there is a major gap in the application of standardized usability assessments to DHTs in MS. Improvements in the standardization of usability assessments will have implications for the future of digital health care for people with MS.

## Introduction

Digital health technologies (DHTs) offer complementary methods to track and manage symptoms, improve treatment adherence, and increase access to healthcare for diverse patient populations^1,2^. In the context of a heterogeneous and prognostically challenging neurodegenerative disorder such as multiple sclerosis (MS), DHTs have significant potential to promote disease management and personalized patient care^3,4^. The potential impact of DHTs in MS is even more apparent when one considers the nature of clinical assessments in this population. Specifically, evaluations typically occur at 6-12-month intervals and require clinical visits for a comprehensive examination. This low frequency of patient consultation is reported to contribute to un/under-reported disease progression^5^. A potential solution is increasing the frequency of clinical consultations, but this is constrained by time, cost, and geography, leading to inequity in healthcare access. To address some of these gaps in care provision, DHTs have become more integrated into the long-term care of people with MS^6–8^.

While the need for innovative digital solutions in MS care is clear, a recent review of 30 unique mobile health applications found that they did not meet the needs of people with MS^9^. Several factors may potentially thwart the successful implementation of DHTs, including but not limited to, the level of digital and health literacy of end users and the perceived usefulness of, or satisfaction with, a DHT^10^. Indeed, it is well established that the incorporation of usability principles into the design and evaluation of DHTs is fundamental to ensuring they are appropriately targeted to the end users’ needs and adopted long-term^11–13^. Usability describes the extent to which DHTs can be “used by specified users to achieve specified goals with effectiveness, efficiency, and satisfaction in a specified context of use”^14^, and typically considers core principles such as effectiveness, learnability, physical comfort/acceptance, ease of maintenance/repairability, and operability^15^. Indeed, the evaluation of usability is important in the process of development and commercialization of DHTs. Government authorities such as the Federal Drug Administration recommend that medical devices (including DHTs) not yet on the market provide a report summarizing potential target users, training necessary for the operation of the product, usability testing, and any problems encountered during technology evaluation^16^. Furthermore, the National Health Service in the UK requires consideration of usability and accessibility principles when approving health apps solicited from industry^17^. Despite such a clear need for usability evaluation of DHTs, there has yet to be a comprehensive assessment of DHT usability in the context of MS. Other groups have assessed the usability of mobile health applications^1,18^, but there is a need to conduct a comprehensive usability evaluation of all DHTs employed by people with MS, especially wearable technologies. The aim of this scoping review was, therefore, to examine the extent to which usability principles have been considered in DHTs for people with MS and to summarize the methods of usability evaluation. A preliminary search of the Joanna Briggs Institute Database of Systematic Reviews and Implementation Reports was conducted to confirm that there are no current or in-progress scoping or systematic reviews on the same topic.

By following a Population Concept Context mnemonic, we generated a primary review question: how has usability been considered in studies of DHTs targeting people with MS? This primary review question was then divided into four sub-questions:

1. What are the participant characteristics (e.g., age, gender, disease severity) included in studies of DHTs in the context of MS?
2. What are the components (e.g., type of technology and delivery platform, development stage) of DHTs targeting people with MS?
3. What assessment methods (e.g., questionnaires, interviews) of usability are incorporated into the design and/or evaluation of DHTs for people with MS?
4. What usability outcomes (e.g., accessibility, flexibility) are reported from the evaluation of DHTs for people with MS?

## Results

### Selection of sources of evidence

**Fig. 1:**
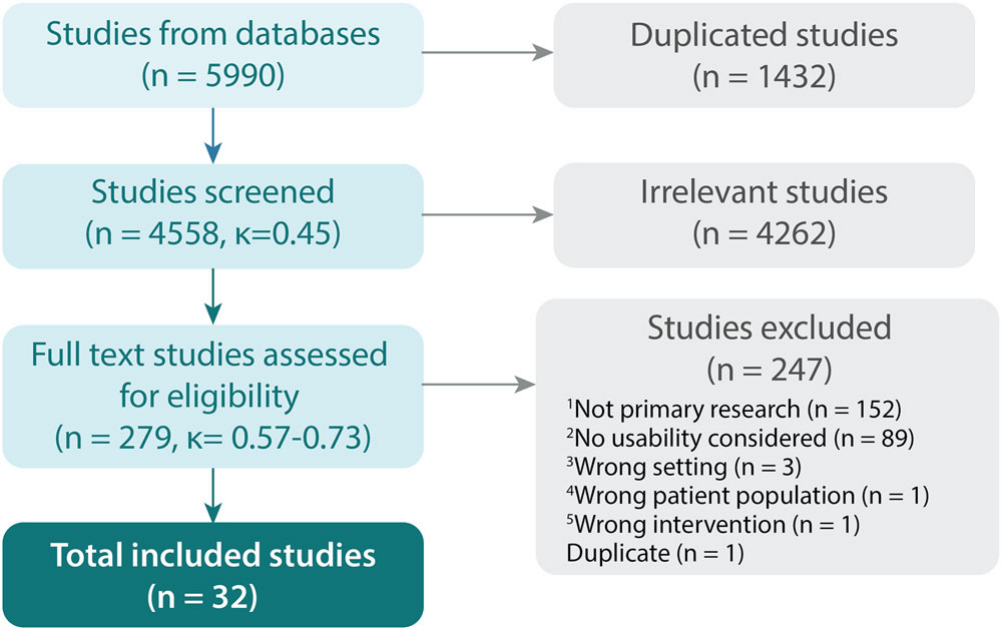
Study selection flow diagram. Data extracted from Covidence. Exclusion Criteria: 1. Review papers, animal studies, unpublished trial data, conference abstracts, opinion pieces, case studies, and letters.; 2. No reported usability outcome measure; 3. Studies with data in MS patients that is not accessible, or that is presented in conjunction with co-morbidities; 4. DHT not for patient populations with MS (primary diagnosis), healthcare practitioners, formal care providers, and researchers; 5. Machine learning and AI studies to assess healthcare data for MS. κ = Cohen’s kappa value.

A total of 5990 studies were identified in the search process (see Fig. 1). Following duplicate removal (*n* = 1432), the titles and abstracts of 4558 studies were screened, and 4262 studies were deemed irrelevant. A total of 279 studies moved to full-text review, where 247 articles were excluded; of note, 89 studies did not assess usability. Inter-rater agreement for title and abstract screening was 0.45, and between 0.57 and 0.73 for full-text review (given there were three raters for this stage). These values indicate moderate to substantial agreement between reviewers, respectively^19^. A total of 32 studies were included in the final narrative synthesis.

### Description of Included Studies and Participants

The characteristics of the included studies and participants are summarized in Table 1. Most studies were conducted in Europe (*n* = 18, 56%), followed by North America (*n* = 9, 28%). Four studies (13%) involved multi-country investigations. More than half of the studies were published between 2020-2023 (n = 19, 59%). Most studies used a mixed-methods (*n* = 16, 50%) or quantitative (*n* = 15, 47%) design. One study (0.3%) used a qualitative design.

**Table 1:** Characteristics of included studies and participants

Ref.	Country	Total sample	Sex (%F)	MS Phenotype (%)	Age Mean/Median (SD/IQR)	EDSS Mean/Median (SD/IQR)	Disease duration Mean/Median (SD/IQR)
Mixed methods (*n* = 16)							
Babbage 2019	UK and NZ	11	55	NR	41–59 (NR)	NR	1.0–10.0 (NR)
Halstead 2020	US	31	81	RRMS: 93 SPMS: 3 PPMS: NR CIS/RIS: NR RPMS: 3	NR	NR	13.2 (NR)
Hsieh 2021^22^	US	10	70	RRMS: 80 SPMS: 10 PPMS: 10 CIS/RIS: 0 RPMS: 0	53.9 (10.9)	2.75 (2.5–6)	15.1 (7.5)
Krause 2022	Germany	23	65	RRMS: 65 SPMS: 21 PPMS:13 CIS/RIS: 0 RPMS: 0	50.0 (24-57)	2.0 (1.0–7)	8.0 (1.5–22.0)
Maillart 2020	France	116	61	RRMS: 74 SPMS: NR PPMS: 25 CIS/RIS: NR RPMS: NR	46.0 (10)	3.6 (1.6)	1.0–11.0 (6.0–30)
Midaglia 2019	US and Spain	76	70	RRMS: 90 SPMS: 5 PPMS: 4 CIS/RIS: NR RPMS: NR	39.5 (7.9)	2.4 (1.4)	11.3 (7.0)
Minen 2020	US	62	89	NR	39.5 (11.6)	NR	NR
Newland 2016	US	7	71	NR	50.7 (9.2)	5 (4.5–6.0)	12.2 (8.2)
Ouwerkerk 2022	NL	77	79	RRMS: 61 SPMS: 18 PPMS: 12 CIS/RIS: NR RPMS: NR	51.1 (10.4)	NR	11.5 (0-39.0)
Palotai 2021	US	64	84	RRMS: 91 SPMS: 7 PPMS: 0 CIS/RIS: 1 RPMS: 0	52.0 (9.0)	2.2 (1.5)	20.0 (8.0)
Rhodes 2019	US	60	65	RRMS: 68 SPMS: 10 PPMS: 3 CIS/RIS: NR RPMS: 6	41.4 (13.3)	0-7 (NA)	8.1 (7.4)
Thirumalai 2018	US	21	67	NR	54.0 (10.8)	NR	NR
Thomas 2021^21^	UK	11	64	RRMS: 72 SPMS: 9 PPMS: 18 CIS/RIS: 0 RPMS: 0	49.0 (8.4)	6.8 (2.5)	1.0–20.0 (NR)
Tonheim & Babic 2018	Norway	4	75	NR	NR	NR	NR
van Kessel 2021	UK and NZ	12	58	NR	47.3 (5.4)	NR	10.9 (4.8)
van Oirschot 2021	NL	25	92	RRMS: 100	41.5 (8.0)	3.1 (1.4)	6.0 (4.4)
Quantitative (*n* = 15)							
Bevens 2022	US CA AUS NZ	15	73	RRMS: 73 SPMS: 6 PPMS: 13 CIS/RIS: NR RPMS: NR	52.0 (11.5)	5 (NR)	12.0 (10.3)
Bove 2019	US	21	86	RRMS: 71 SPMS: 9 PPMS: 19 CIS/RIS: 0 RPMS: 0	53.8 (11.6)	3.1 (2)	14.5 (9.6)
D’hooghe 2018	Belgium	75	67	RRMS: 100	39.2 (10.1)	2 (1.5-3)	NR
D’Arma 2022	Italy	37	41	RRMS: 48 SPMS: 51 PPMS: 0 CIS/RIS: 0 RPMS: 0	48.0 (8.6)	5.5 (1.8)	17.0 (8.1)
Defer 2018	France	91	81	RRMS: 100	38.0 (30.5)	1 (0.0–2.5)	NR
Fernandez-Vazquez 2021	Spain	40	53	RRMS: 23 SPMS: 50 PPMS: 28 CIS/RIS: 0 RPMS: 0	49.5 (7.9)	6.4 (1.5)	NR
Finkelstein and Jiazhen 2018	US	10	NR	NR	55.0 (10)	NR	24.0 (12.0)
Jonsdottir 2018	Italy	16	75	NR	56.8 (12.3)	6.5 (6.5–7.0)	19.4 (12.3)
Mokhberdezful 2021	Iran	126	77.5	NR	36.8 (10.3)	NR	NR
Nasseri 2020	Germany	19	47	NR	51.1 (7.9)	3.5 (2.7–6.0)	16.5 (9.3)
Pagliari 2021	Italy	30	60	NR	48.3 (9.6)	5 (3.5-6)	12.7 (6.7)
Stuart 2020	UK	56	54	RRMS: NR SPMS: 42 PPMS: 57 CIS/RIS: NR RPMS: NR	53.6 (8)	5.7 (1.3)	12.2 (8.6)
Tacchino 2015	Italy	16	81	RRMS: 56 SPMS: 44 PPMS: 0 CIS/RIS: 0 RPMS: 0	49.1 (9.1)	3.7 (1.9)	13.5 (9.1)
van Beek 2020	Switzerland	9	100	RRMS: 55 SPMS: 22 PPMS: 22 CIS/RIS: NR RPMS: NR	53.9 (12.3)	3.9 (1.9)	10.6 (9.7)
Woelfle 2023	Switzerland	31	68	RRMS: 74, SPMS: 6 PPMS: 12, CIS/RIS: 6 RPMS: NR	43.4 (12)	3 (1.0–6)	NR
Qualitative (n = 1)							
Dennett 2020	UK	11	91	RRMS: 36 SPMS: 54 PPMS: 9 CIS/RIS: 0 RPMS: 0	28-68 (NR)	4.0–6.5 (NR)	1.0–40.0 (NR)

*AUS* Australia, *CIS* Clinically Isolated Syndrome, *EDSS* Expanded Disability Status Scale, *DHT* Digital Health Technology, *IQR* Interquartile Range, *MS* Multiple Sclerosis, *NL* Netherlands, *NR* Not reported, *NZ* New Zealand, *PPMS* Primary Progressive MS, *RIS* Radiologically Isolated Syndrome, *RPMS* Relapsing Progressive MS, *RRMS* Relapsing Remitting MS, *SD* Standard Deviation, *SPMS* Secondary Progressive MS, *UK* United Kingdom, *US* United States, *VR* Virtual Reality.

Across the studies, the total number of participants was 1213, with the sample sizes ranging between 4 and 126. Most participants were female (71%), with RRMS (73%). Two studies (6.3%) included people with CIS. Of the 28 (86%) studies that reported mean/median age of participants, the age ranged between 36.8 and 56.8 years old. Across all participants, the mean EDSS scores ranged between 1 and 6.5, while the mean disease duration ranged between 6 and 24 years. The education level of participants was reported in 11 (34%) studies and ranged between 8^th^ grade and doctorate level.

### Description of Usability Components of Digital Health Technologies (DHTs)

The characteristics of the DHTs are summarized in Table 2. The majority of DHTs were application-based (apps) (*n* = 23, 70%), followed by wearables (*n* = 7, 21%), Website/Internet (*n* = 6, 18%), and others (n = 2, 6%): a game console and a virtual reality system. Five studies used a combination of apps and wearables (*n* = 4) or apps and Internet/Website (*n* = 1). Most DHTs were implemented in the patient’s home or community (*n* = 30, 94%). The remaining two DHTs (6%) were used in a hospital/clinic setting. Of the 30 DHTs implemented in a patient’s home, eight were additionally evaluated within a research facility (*n* = 7) or hospital (*n* = 1). All DHTs were evaluated by people with MS (*n* = 32), with seven DHTs evaluated by two users (22%): people with MS and formal health/social care providers (*n* = 6) or family caregivers (*n* = 1). Over half the DHTs were the final versions (*n* = 17, 53%), while the remainder were in various stages of iterative development (*n* = 15, 47%). The DHTs were intended for various uses, including remote self-management, education, symptom assessment and monitoring, cognitive and physical rehabilitation, and supporting therapeutic interventions.

**Table 2:** Characteristics of DHTs, usability and testing methods across included studies

Ref.	Type of DHT	Name & Purpose of DHT	Implementation Setting	Evaluator(s)	Stage & Model of Development	Method of evaluation	Evaluation tools	Usability characteristics
Mixed methods (*n* = 16)								
Babbage 2019	App	MS Energize: iPhone app focused on self-management of fatigue using CBT principles	Home/Community	Patients	Iterative	Questionnaires Interviews	SUS	Satisfaction Learnability
Halstead 2020	Website/ Internet	A telehealth resilience-based skills intervention	Home/Community	Patients Caregivers	Final version	Questionnaires	Independent rating scales	Engagement Satisfaction
Hsieh 2021^22^	App	Steady MS: A mobile application for assessing fall risk.	Home/Community Research facility	Patients	Iterative	Questionnaires Task completion Interviews	SUS Other: MDPQ	Efficiency Intuitive Proficiency Satisfaction Usefulness
Krause 2022	App	Levidex: A adaptable digital lifestyle management toolkit	Home/Community	Patients Health/social care providers	Iterative	Questionnaires Interviews	Independent rating scales	Acceptability Practicality Satisfaction
Maillart 2020	App	MSCopilot: A medical software device for the remote self-assessment	Home/Community	Patients Health/social care providers	Final version	Questionnaires Other: Descriptive analysis	Independent rating scales	Satisfaction
Midaglia 2019	Wearables App	Smartphone/watch-based self-administered assessment tool for hand motor function, gait and posture, mood, and cognitive impairment	Home/Community	Patients	Iterative	Questionnaires Interviews	Independent rating scales	Adherence Satisfaction
Minen 2020	App	RELAXaHEAD: A mobile app to assess and monitor headaches	Home/Community	Patients	Final version	Questionnaires Interviews Focus groups	Independent rating scales	Acceptability Adherence Engagement Feasibility Retention
Newland 2016	Wearables	GAITRite Kinect Gait Sensor: An in-home electronic gait monitoring system	Home/Community Research facility	Patients	Iterative	Interviews	N/A	Acceptability Feasibility
Ouwerkerk 2022	App	Whereabouts: A remote mobile application to assess societal participation	Home/Community	Patients	Iterative	Questionnaires Task completion Other: App data analysis	Independent rating scales	Effectiveness Efficiency Satisfaction
Palotai 2021	App	A mobile application for circadian assessment and differentiation of fatigue phenotypes and other mood symptoms	Home/Community	Patients	Final version	Task completion	N/A	Adherence Compliance
Rhodes 2019	App	MS Performance Test (MPT): A mobile tablet-based assessment program for remote self-assessment	Hospital/Clinic	Patients Health/social care providers	Iterative	Questionnaires Task completion	Independent rating scales Other: Open-text responses	Completion rate/time Satisfaction
Thirumalai 2018	App	Tele-Exercise and Multiple Sclerosis (TEAMS): An at-home tele-exercise program comprising a computer, tablet and an adjustable floor stand	Home/Community	Patients	Iterative	Questionnaires Task completion Interviews Focus groups Think aloud protocol Heuristic testing	Other: Usability Problem Taxonomy	Effectiveness Satisfaction Usefulness
Thomas 2021^21^	App	A digital homework toolkit for the Fatigue: Applying Cognitive Behavioral and Energy Effectiveness Techniques to Lifestyle (FACETS) program: a 6-session group fatigue management program	Home/Community Research facility	Patients	Iterative	Questionnaires Interviews Focus groups Task completion	SUS	Design Content functionality Learnability Satisfaction
Tonheim and Babic 2018	App	msHelse: A mobile application for symptoms self-management	Home/Community Research facility	Patients	Iterative	Questionnaires; Interviews	SUS	Satisfaction
van Kessel 2021	App	MS Energize: A mobile application for self-management of fatigue management	Home/Community	Patients	Iterative	Questionnaires; Interviews; Task completion	SUS	Accessibility Usability
van Oirschot 2021	Wearables App Website /Internet	MS Sherpa: mobile application & wearable for remote monitoring of walking speed	Home/Community	Patients	Final version	Task completion; Interviews	N/A	Learnability Usability
Qualitative (n = 15)								
Bevens 2022	Website /Internet	Multiple Sclerosis Online Course (MSOC): A web-based educational lifestyle program	Home/Community	Patients	Iterative	Questionnaires	Independent rating scales	Acceptability Accessibility Desirability Learnability
Bove 2019	App Website/ Internet	EVO^TM^: A digital videogame-based treatment designed to improve attention and inhibitory control	Home/Community	Patients	Iterative	Task completion	N/A	Adherence Feasibility
D’hooghe 2018	App	VIOLA: A web-based mobile application for maintaining progress following a multidisciplinary rehabilitation program	Home/Community	Patients	Iterative	Questionnaires	Independent rating scales	Ease-of-use Likeability Satisfaction Usefulness
D’Arma 2022	Wearables App	MS TeleCoach: A mobile telerehabilitation application targeting fatigue by coaching and monitoring physical activity	Home/Community	Patients	Final version	Questionnaires	Other: QUEST	Satisfaction
Defer et al. 2018	App	Vigip-SEP: A mobile application targeting improved reporting of adverse drug reactions	Home/Community	Patients Health/social care providers	Final version	Questionnaires	Independent rating scales	Satisfaction
Fernandez-Vazquez 2021	Wearables	KSO GT®exoskeleton: A wearable lower limb exoskeleton for gait rehabilitation	Home/Community Research facility	Patients Health/social care providers	Final version	Questionnaires	Independent rating scale Other: QUEST 2.0 CSQ-8	Satisfaction
Finkelstein and Jiazhen 2018	Website /Internet	Interactive & personalized telerehabilitation program for pwMS	Home/Community	Patients	Final version	Questionnaires Task completion	Independent rating scales	Completion rate/time Ease-of-use Satisfaction
Jonsdottir 2018	Other: Game consoles	REHAB@HOME Kinect: A virtual game-based therapy intervention for improving motor functioning and spatial awareness	Hospital/Clinic	Patients	Final version	Questionnaires	Independent rating scales	Feasibility Motivation Satisfaction User experience
Mokhberdezful 2021	App	A mobile application for MS self-management	Home/Community Hospital/Clinic	Patients Health/social care providers	Final version	Questionnaires	Other: QUIS questionnaire	Satisfaction
Nasseri 2020	App	A mobile app providing evidence-based information on the benefits of physical activity	Home/Community	Patients	Final version	Questionnaires	Independent rating scales	Comprehensibility Usefulness
Pagliari 2021	Other: VR	An at-home virtual-reality rehabilitation system targeting cognitive and motor interventions	Home/Community	Patients	Final version	Questionnaires	SUS	Learnability Satisfaction Usability
Stuart 2020	Wearables	A wearable remote- physiological activity monitoring device for pwMS	Home/Community	Patients	Final version	Task completion Other: Patient feedback Adverse reactions Tolerance (wear time)	N/A	Tolerance (Device wear time) Adverse reactions
Tacchino 2015	App	Cognitive Training Kit (COGNI-TRAcK): A mobile phone and tablet-based app for self-administration of a cognitive rehabilitation intervention based on working memory exercises	Home/Community	Patients	Final version	Questionnaires	Independent rating scales	Compliance Motivation
van Beek 2020	App	A home-based tablet application for dexterity training	Home/Community Research facility	Patients	Final version	Questionnaires	SUS; Other: CUEQ	Adherence Effectiveness Efficiency Satisfaction
Woelfle et al. 2023	Wearables App	dreaMS: A mobile application & sensors for the remote ongoing assessment of MS symptoms	Home/Community Research facility	Patients	Iterative	Questionnaires Other: Mean-daily-wear-fraction	Independent rating scales	Acceptability Meaningfulness
Qualitative (*n* = 1)								
Dennett 2020	Website /Internet	WEBPaMS: A web-based physiotherapy program (www.webbasedphysio.com)	Home/Community	Patients	Final version	Interviews	N/A	Convenience Feasibility Portability

*AMSQ* Arm Function in Multiple Sclerosis Questionnaire, App Application-based, *CSQ-8* Client Satisfaction Questionnaire, *CUEQ* Custom User Engagement Questionnaire, *DHT* Digital Health Technology, *MDPQ* Mobile Device Proficiency Questionnaire, *NR* Not reported, SUS System Usability Scale, *QUEST* Quebec User Evaluation of Satisfaction with Assistive Technology.

### Methods of Usability Evaluation

The methods of usability evaluations within each study are detailed in Table 2. When considering the number of methods used to evaluate DHTs across the studies, there was an even split, wherein half of the studies implemented a single method of evaluation (*n* = 16, 50%), while the remaining studies utilized two (*n* = 9, 28%) or more methods (*n* = 7, 22%). Usability of the DHTs was most commonly evaluated using questionnaires (n = 26, 81%), followed by interviews (*n* = 12, 37%), task completion tests (*n* = 9, 28%), think aloud protocols (*n* = 4, 12%), focus groups (*n* = 3, 9%), and others (*n* = 4, 12%) which included patient feedback. Within the 26 studies that utilized questionnaires, most included scales developed by the research team (*n* = 15, 58%). Of the studies that used a standardized questionnaire, the most common scale was the System Usability Scale (SUS)^20^, used in seven studies (27%).

### Description of Usability Outcomes

Table 2 summarizes the usability characteristics across the included studies. There was a wide variety and number of usability characteristics reported across studies, with 20 unique usability characteristics reported in over a third of studies (*n* = 12, 37.5%). Three studies (9%) reported usability as a general term. Across all studies, the most assessed usability characteristic was user satisfaction (*n* = 17, 53%). Other usability characteristics assessed were adherence (*n* = 6, 19%), acceptability (*n* = 5, 16%), feasibility (*n* = 5, 16%), usefulness (*n* = 4, 12.5%), and efficiency (*n* = 3, 9%). There were seven usability characteristics only reported by two independent studies. Most studies reported two (*n* = 13, 40.5%) or more (*n* = 13, 40.5%) usability characteristics, while six studies (19%) reported a single usability characteristic. While the overall conclusions from usability assessment varied, studies most often reported a combination of positive feedback and suggestions for improving future iterations of the DHTs. Two studies explicitly mentioned that participant feedback from usability assessment was incorporated into DHT development^21,22^.

## Discussion

The current scoping review is the first to examine usability characteristics and testing methods in DHTs with a specific focus on people with MS. Usability was evaluated in less than a third of relevant studies, indicating limited consideration of this important topic within the context of DHTs in MS. Evaluation of usability was highly heterogeneous across studies, both in terms of the number of reported characteristics and assessment methods. The most evaluated DHTs were mobile applications and most studies used different types of questionnaires to assess usability. DHTs were evaluated by people with MS, with limited inclusion of health/social care providers or family caregivers in the process. Below, we first summarize key findings and knowledge gaps, and subsequently make recommendations for future work to advance the design and implementation of DHTs in MS.

DHTs have become a rapidly evolving modern health intervention tool, especially amidst the COVID pandemic, and likely beyond^23^. The importance of evaluating the usability of DHTs has been recently highlighted in the context of other chronic diseases, such as Parkinson’s disease, and in elderly individuals^24–27^. A recent review of mobile applications in MS reported only six of 14 studies had evaluated usability^18^. In the current review, which encompassed the broad spectrum of DHTs, the 32 studies only represented 26% of relevant DHT studies in MS. There is a clear lack of usability assessment in over half of DHT studies in MS. Furthermore, we reported minimal involvement of caregivers and health/social care professionals. The inclusion of usability assessments by formal care providers and family caregivers should be considered given the importance of MS caregivers in patient care^28,29^. Evaluation of usability is a critical part of the development and effective use of DHTs, and therefore should be considered in emerging DHTs targeting people with MS.

Our findings show that the evaluation methods and usability outcomes assessed were very heterogeneous across studies. Indeed, this heterogeneity in usability assessment and lack of consistency across studies have been reported across other neurodegenerative and chronic diseases, such as dementia, diabetes, and cardiovascular disease^24,30^. Usability assessment approaches similarly varied more broadly in studies investigating medical devices across several populations depending on the DHT and its intended use^31^. In the current study, the most frequently used method to evaluate usability was questionnaires, and most studies implemented independent, non-validated, questionnaires developed by the research team. The most commonly used standardized questionnaire was the SUS. Although a valid and reliable measure, the SUS is a self-reported measure with inherent bias and was not intended to be comprehensive in its approach to evaluate usability^32,33^. Further, the SUS is not specific nor adapted to a particular DHT type, limiting its ability to describe specific aspects of usability.

The usability of a DHT can encompass a wide range of characteristics and these outcomes will vary based on the intended use of the DHT. We reported a large number of different usability outcome characteristics across studies, with the most common usability characteristic evaluated being user satisfaction. Indeed, the SUS questionnaire, the most used usability assessment method, primarily captures user satisfaction, with additional sub-scales for learnability and usability. The usability characteristics reported varied across studies but were somewhat consistent within DHT type. For example, DHT studies that incorporated a wearable component assessed tolerance or wear-time, whereas self-management DHTs used terms such as engagement and acceptability. Furthermore, there were a large number of studies that reported unique descriptors of usability. It is important to capture multiple characteristics of usability, however consistent terminology and descriptions of usability outcomes is important for cross-study comparisons and validation. There is a clear need to develop more standardized and comprehensive approaches to assess the usability of DHTs for people with MS.

The dramatic rise in remote patient management necessitates a framework to effectively evaluate the intended use and quality of DHTs to enhance and optimize user experience. Herein, we provide recommendations to advance research on DHTs in MS. Usability assessments and outcomes should be tailored for the intended use of the DHT and the target user. Usability testing does not require an enormous time investment, in fact, the likelihood of acquiring novel information after six to nine users is minimal^34^. We found an average of 35 participants assessed usability across the studies included in this review, which is indeed sufficient to reliably and effectively assess DHT usability. Half the evaluated studies were still undergoing development of their DHT, and only two studies explicitly reported that participant feedback from usability testing was incorporated into subsequent DHT development^21^. Future studies should also consider usability in the context of people with MS who experience additional barriers when accessing DHTs, such as low socio-economic status, rurality, older age, and more severe disability^35^.

The future of DHTs in MS requires an updated standardized usability framework, and more targeted usability outcomes specific to the type and application of DHT in patient care. Implementation of both qualitative and quantitative measures is important^36,37^. To update currently used standardized methods, like the SUS, objective and comprehensive measures of usability assessments are needed that do not rely only on self-report. Future work should therefore apply a mixed methods approach to assess usability and implement user feedback during stages of DHT development. Incorporating these considerations, we recommend the following to improve the assessment of DHT usability:

1. Common and clearly defined usability characteristics of DHTs should be evaluated. For example, user satisfaction and acceptability characteristics could be rated on a numeric scale.
2. Additional criterion specific to the type of DHT, and user (patient or caregiver) should be assessed separately. For example, wearables should include wear-time, and apps that require tests should include task completion time.
3. Qualitative measures, such as interviews or focus groups, should be conducted in conjunction with quantitative measures. These should assess user feedback and aim to report the subtle challenges with usability not captured by quantitative measures.

These usability metrics, if combined in the form of a summative score, could be useful to compare across studies of various DHT types. Usability results from these metrics should be integrated into the development of the DHT. Finally, the current scoping review highlights a major gap in the application of standardized usability evaluations and outcomes of current DHTs implemented in MS care. Importantly, our results highlight the opportunity to implement improved methods of usability assessment which will have major implications in the future of mobile care for people living with MS.

This review has several limitations that warrant consideration. First, we have included only English-language articles due to a lack of resources for translation. It is possible that articles published in other languages may have included additional information on usability evaluations of DHTs designed for people with MS. Telehealth and virtual telerehabilitation studies were also excluded in the current review. Furthermore, given the heterogeneity in usability principles and methods used to evaluate usability, it was not possible to synthesize the quantitative and qualitative data reported accurately. Nonetheless, we extracted and included these data in Supplementary Fig. 4 for interested readers. The usability of most DHTs was reported to be good, with satisfaction ranging from ~80-90%. The lowest usability scores were typically associated with wearable technologies. Few studies further evaluated the 10-20% of pwMS who struggled with DHT usage. Future research should focus on this sub-group of the MS population to understand how to develop more targeted and usable DHTs.

## Methods

We followed the Joanna Briggs Institute guidance for scoping reviews^38,39^. The review is reported in accordance with the PRISMA Extension for Scoping Reviews^40^ (Supplementary Fig. 1). We have registered our protocol prospectively in the Open Science Framework: https://osf.io/y7gqp/.

### Eligibility Criteria

#### Participants

Studies focusing on adults (≥18 years old) with MS and/or their caregivers were considered for inclusion. Any subtype of MS, including Clinically/Radiologically Isolated Syndrome (CIS/RIS), Relapsing-Remitting MS (RRMS), Primary Progressive MS (PPMS), and Secondary Progressive MS (SPMS) was eligible for inclusion. We excluded animal studies and studies involving mixed populations (i.e., MS and other conditions) where data from people with MS could not be separated from other conditions. We further excluded studies focusing on formal health and/or social care professionals.

#### Concept

Studies that described usability characteristics (e.g., comfort, ease of use, accessibility, flexibility, etc.) and/or usability testing methods (e.g., questionnaires, task completion, “Think-Aloud” protocols, interviews, heuristic testing, and focus groups, etc.) of DHTs were included. We excluded multicomponent studies in which data on the DHT component could not be extracted. Studies on electronic medical records, medical monitoring devices, machine learning, artificial intelligence, biomedical applications, systems for intelligent processing of genetic data, and assistive devices, were excluded.

#### Context

We considered studies conducted in any geographic location and setting (e.g., hospital settings, primary care, community care, or at home) and published in English. Our pre-screening results found limited studies on DHTs in the context of MS prior to 2010. Therefore, to focus on the most recent and relevant DHT studies, we considered studies published from 2010 until the present.

#### Types of Sources

We included peer-reviewed quantitative, qualitative, and mixed-methods studies. We recognize that computer-based digital technology development may be conducted outside of academia and published in non-traditional or non-peer-reviewed outlets – but in the context of providing evidence-based information for health and social care providers and researchers, peer-reviewed evidence is considered the gold standard. We excluded systematic and non-systematic reviews, dissertations, conference abstracts and proceedings, observational studies, case reports, opinion pieces, commentaries, and protocols.

### Search Strategy

We implemented a systematic, peer-reviewed three-step search strategy in line with the framework developed by the Joanna Briggs Institute and in consultation with a health sciences librarian. The process began with a preliminary search in PubMed to find key articles relevant to the three components of the research question: usability, DHTs, and MS. Using these articles, a list of keywords was developed for the search strategy, and the syntax was modified such that it could be applied to the other databases. The systematic search was then run in five databases: Web of Science, OVID Medline, CINAHL, Embase, and medRxiv. Specific to the medRxiv search, only the MS search component was used. An example of the search terms used in Medline can be found in Supplementary Fig. 2.

### Selection of Sources of Evidence

All results were uploaded to Covidence (Veritas Health Innovation, Melbourne, Australia) to facilitate de-duplication, screening of titles and abstracts, full-text review, and extraction. Titles and abstract screening were pilot-tested by two reviewers on a random sample of 10 studies before screening. Following pilot testing, all authors were involved in both screening phases, with two independent reviewers examining each article. When reviewers disagreed about the inclusion status of a citation, another reviewer examined the citation, and a three-way discussion was held to reach a consensus. Full texts for all potentially relevant articles were uploaded to Covidence for further screening by three independent reviewers. Discrepancies in the inclusion/exclusion of full-text studies were resolved during a consensus meeting. A manual search of reference lists from the full-text articles included was then performed to identify additional studies that met the inclusion criteria.

### Data Charting Process

Data extraction was completed using the Covidence 2.0 customizable template with categories adapted from the Joanna Briggs Institute^38^. Extracted variables included study characteristics (authors, year of publication, country of origin, study design), participant characteristics (age, sex, disease duration, Expanded Disability Status Scale (EDSS), and MS phenotype), DHT information (name, type, purpose, implementation setting, stage of DHT development) and usability considerations (evaluation method, questionnaire type, and DHT evaluator). We piloted the template, which led to the inclusion of “not reported” options for several items and the addition of examples to some item definitions to enhance consistency and ease of use. Three independent reviewers performed data extraction. When reviewers disagreed about the inclusion status of a citation, another reviewer examined the citation, and a three-way discussion was held to reach a consensus.

### Data analysis

Data synthesis was performed in Microsoft Excel (Microsoft Office, 2019). We calculated inter-rater agreement during title and abstract and full-text screening (before the consensus meeting) using Cohen’s Kappa. No formal measures of agreement were used during the data extraction because differences in capitalization and punctuation generated messages of inconsistency, even if the critical content was the same between reviewers. Our focus was on the synthesis of descriptive features of the studies relative to usability, not on a synthesis of actual study results. We used descriptive statistics (frequencies, median and ranges), with data presented graphically and in tabular format as appropriate. We generated descriptive summaries of study characteristics (i.e., frequency/distribution of publication year, country in which the study was conducted, study design), participant characteristics (mean/median age, disease duration, and Expanded Disability Status Scale (EDSS), and frequency/distribution of sex, and MS phenotype), DHTs included in the literature (frequency/distribution of type, implementation setting, stage of DHT development), and usability considerations (frequency/distribution of usability characteristics, testing methods, and usability evaluator).

### Reporting summary

Further information on research design is available in the Nature Research Reporting Summary linked to this article.

### Supplementary information

Supplementary Information

Reporting Summary

## Data Availability

No data sets were generated or analyzed during the current study. The aggregated data analyzed in this study are available from the corresponding author upon reasonable request. This scoping review uses peer-reviewed articles and therefore does not require ethical approval.

## Code availability

No code was generated during the current study.
