# Supplementary material for: A scoping review assessing the usability of digital health technologies targeting people with multiple sclerosis
Source: NPJ Digit Med. 2024 Jun 25;7:168. doi: 10.1038/s41746-024-01162-0 (PMC11199563; doi:10.1038/s41746-024-01162-0)
Supplement: Supplementary file 1 — Supplementary Information [file 41746_2024_1162_MOESM1_ESM.pdf]

Supplementary Figure 1. Preferred Reporting Items for Systematic reviews and Meta-Analyses extension for Scoping Reviews (PRISMA-ScR) Checklist

SECTION	ITEM	PRISMA-ScR CHECKLIST ITEM	REPORTED ON PAGE #
**TITLE**			
Title	1	Identify the report as a scoping review.	1
**ABSTRACT**			
Structured summary	2	Provide a structured summary that includes (as applicable): background, objectives, eligibility criteria, sources of evidence, charting methods, results, and conclusions that relate to the review questions and objectives.	2
**INTRODUCTION**			
Rationale	3	Describe the rationale for the review in the context of what is already known. Explain why the review questions/objectives lend themselves to a scoping review approach.	3
Objectives	4	Provide an explicit statement of the questions and objectives being addressed with reference to their key elements (e.g., population or participants, concepts, and context) or other relevant key elements used to conceptualize the review questions and/or objectives.	4
**METHODS**			
Protocol and registration	5	Indicate whether a review protocol exists; state if and where it can be accessed (e.g., a Web address); and if available, provide registration information, including the registration number.	5
Eligibility criteria	6	Specify characteristics of the sources of evidence used as eligibility criteria (e.g., years considered, language, and publication status), and provide a rationale.	6
Information sources*	7	Describe all information sources in the search (e.g., databases with dates of coverage and contact with authors to identify additional sources), as well as the date the most recent search was executed.	6
Search	8	Present the full electronic search strategy for at least 1 database, including any limits used, such that it could be repeated.	Supp Fig 1
Selection of sources of evidence†	9	State the process for selecting sources of evidence (i.e., screening and eligibility) included in the scoping review.	7
Data charting process‡	10	Describe the methods of charting data from the included sources of evidence (e.g., calibrated forms or forms that have been tested by the team before their use, and whether data charting was done independently or in duplicate) and any processes for obtaining and confirming data from investigators.	7
Data items	11	List and define all variables for which data were sought and any assumptions and simplifications made.	6
Critical appraisal of individual sources of evidence§	12	If done, provide a rationale for conducting a critical appraisal of included sources of evidence; describe the methods used and how this information was used in any data synthesis (if appropriate).	N/A
Synthesis of results	13	Describe the methods of handling and summarizing the data that were charted.	8

SECTION	ITEM	PRISMA-ScR CHECKLIST ITEM	REPORTED ON PAGE #
**RESULTS**			
Selection of sources of evidence	14	Give numbers of sources of evidence screened, assessed for eligibility, and included in the review, with reasons for exclusions at each stage, ideally using a flow diagram.	8
Characteristics of sources of evidence	15	For each source of evidence, present characteristics for which data were charted and provide the citations.	Table 1 and Table 2
Critical appraisal within sources of evidence	16	If done, present data on critical appraisal of included sources of evidence (see item 12).	N/A
Results of individual sources of evidence	17	For each included source of evidence, present the relevant data that were charted that relate to the review questions and objectives.	9-11
Synthesis of results	18	Summarize and/or present the charting results as they relate to the review questions and objectives.	9-11
**DISCUSSION**			
Summary of evidence	19	Summarize the main results (including an overview of concepts, themes, and types of evidence available), link to the review questions and objectives, and consider the relevance to key groups.	11-12
Limitations	20	Discuss the limitations of the scoping review process.	15
Conclusions	21	Provide a general interpretation of the results with respect to the review questions and objectives, as well as potential implications and/or next steps.	15
**FUNDING**			
Funding	22	Describe sources of funding for the included sources of evidence, as well as sources of funding for the scoping review. Describe the role of the funders of the scoping review.	21

JBI = Joanna Briggs Institute; PRISMA-ScR = Preferred Reporting Items for Systematic reviews and Meta-Analyses extension for Scoping Reviews.

* Where *sources of evidence* (see second footnote) are compiled from, such as bibliographic databases, social media platforms, and Web sites.

† A more inclusive/heterogeneous term used to account for the different types of evidence or data sources (e.g., quantitative and/or qualitative research, expert opinion, and policy documents) that may be eligible in a scoping review as opposed to only studies. This is not to be confused with *information sources* (see first footnote).

‡ The frameworks by Arksey and O'Malley (6) and Levac and colleagues (7) and the JBI guidance (4, 5) refer to the process of data extraction in a scoping review as data charting.

§ The process of systematically examining research evidence to assess its validity, results, and relevance before using it to inform a decision. This term is used for items 12 and 19 instead of "risk of bias" (which is more applicable to systematic reviews of interventions) to include and acknowledge the various sources of evidence that may be used in a scoping review (e.g., quantitative and/or qualitative research, expert opinion, and policy document).

From: Tricco AC, Lillie E, Zarin W, O'Brien KK, Colquhoun H, Levac D, et al. PRISMA Extension for Scoping Reviews (PRISMAScR): Checklist and Explanation. Ann Intern Med. 2018;169:467–473. doi: 10.7326/M18-0850.

Supplementary Figure 2. Representative Search Strategy (Web of Science)

Theme	Search Terms
multiple sclerosis	**"multiple sclerosis"** (Topic) or **"clinically isolated syndrome"** (Topic) or **"relapsing remitting"** (Topic) or **"secondary progressive"** (Topic) or **"primary progressive"** (Topic)
AND digital health technology	**"mobile applications"** (Topic) or **smartphone** (Topic) or **"text messaging"** (Topic) or **telemedicine** (Topic) or **telerehabilitation** (Topic) or **"wearable electronic device*"** (Topic) or **"fitness tracker*"** (Topic) or **"medical electronics"** (Topic) or **"cell phone*"** (Topic) or **cellphone*** (Topic) or **"smart phone*"** (Topic) or **smartphone*** (Topic) or **"tele health"** (Topic) or **telehealth** (Topic) or **telemonitoring** (Topic) or **"tele monitoring"** (Topic) or **"mobile technolog*"** (Topic) or **"mobile app*"** (Topic) or **"i phone*"** (Topic) or **iphone*** (Topic) or **"mobile phone*"** (Topic) or **android** (Topic) or **"cellular phone*"** (Topic) or **tablet*** (Topic) or **wireless*** (Topic) or **"tele medicine"** (Topic) or **telemedicine** (Topic) or **"tele rehabilitation"** (Topic) or **mhealth** (Topic) or **"m health"** (Topic) or **mobile** (Topic) or **"text messag*"** (Topic) or **"direct messag*"** (Topic) or **"SMS messag*"** (Topic) or **"short message servic*"** (Topic) or **"smart messag*"** (Topic) or **ehealth** (Topic) or **"e health"** (Topic) or **"electronic health"** (Topic) or **"phone app*"** (Topic) or **ipad** (Topic) or **"i pad"** (Topic) or **"e therapy"** (Topic) or **etherapy** (Topic) or **SMS** (Topic) or **wearable*** (Topic) or **"u health"** (Topic) or **uhealth** (Topic) or **"electronic sensor"** (Topic) or **"i pod*"** (Topic) or **ipod*** (Topic) or **"e monitor*"** (Topic) or **"electronic monitor*"** (Topic) or **"smart watch*"** (Topic) or **"smartwatch*"** (Topic)

Additional search details: Limited to English language primary research articles (including early access); total article yield: 1,226. Duplicates with searches from other databases were removed upon upload to Covidence.

Supplementary Figure 3: Data Extraction Template

1. **Citation** (include the first author's last name and year of publication; if more than 2 authors use et al. – e.g., Adamson et al. 2016)

2. **Journal name** (in full)

3. **Year of publication**
   2016
   2017
   2018
   2019
   2020
   2021
   2022
   2023

4. **Title**

5. **Study design**
   Quantitative
   Qualitative
   Mixed methods
   Other __________

6. **Country in which the study was conducted** (if other, please indicate country in the text box)

US	Germany
UK	Sweden
Canada	Finland
Australia	China
Italy	Norway
Belgium	New Zealand
Netherlands	Spain
Denmark	Turkey
Italy	Iran
Sweden	Israel
Ireland	Other __________

7. **Total sample size**

8. **Number of female participants**

9. **Inclusion criteria**

10. Exclusion criteria

11. Mean/median age

12. SD/IQR age

13. Mean/median EDSS/PDDS

14. SD/IQR EDSS/PDDS

15. RRMS (Number of participants with RRMS phenotype)

16. RPMS (Number of participants with RPMS phenotype)

17. PPMS (Number of participants with PPMS phenotype)

18. SPMS (Number of participants with SPMS phenotype)

19. CIS/RIS (Number of participants with CIS)

20. Mean/median disease duration

21. SD/IQR disease duration

22. Highest Education level

- 8th grade
- Technical/Trade school
- Bachelor's degree
- Master's degree
- Doctorate
- Education level not reported
- Other __________

23. Name of the DHT

24. Implementation setting

- Home or community
- Hospital or clinic
- Research facility
- Other __________

25. Type of DHT

- Wearables
- App
- Website/internet

Other __________

26. Evaluation method(s) for usability

Questionnaires
Task completion
Interviews
Focus groups
Think aloud protocol
Heuristic testing
Not reported
Other __________

27. What questionnaire was used?

System Usability Scale
Post-Study System Usability Questionnaire
Technology Acceptance Model Questionnaire
Task Index
AdEQUATE (questionnAire for Evaluation of QUAlity in TElemedicine systems)
Not applicable - a questionnaire was not used
Rating scales developed by the research team
Other __________

28. Who evaluated the DHT?

Patients
Health and/or social care professionals
Caregivers
Heuristics experts
Not reported

29. Iterative model of development (we want to see if any of the articles mentioned the development of further iterations of the app as a result of the usability testing)

Non-Iterative
Iterative
Other __________

Supplementary Figure 4: Summary of usability testing results for persons with MS.

Ref.	Year	DHT Purpose	Usability Assessment Method	Usability Testing Results
Babbage et al.	2019	MS Energize: iPhone app focused on self-management of fatigue for pw MS using CBT principles.	Questionnaire and qualitative interviews.	High SUS score (75), 4 themes emerged: Validation, personal cost, adding to knowledge, and a good idea—for someone.
Bevens et al.	2022	Multiple Sclerosis Online Course (MSOC): A web-based educational lifestyle program for pwMS.	Program Acceptability, Accessibility, Learnability, Desirability	The course was fully completed by 60% of participants. 12 found it easy and 4 found it difficult to login. Most participants found navigating the modules easy. 15 found the course easy to learn while 5 required assistance from researchers. All content types were well received.
d'Arma et al.	2021	Virtual Instrument fOr healthy Lifestyle Adherence (VIOLA): A web-based educational application targeting behaviour and lifestyle issues for pwMS.	Satisfaction questionnaire	Most participants (89%) responded to the questionnaire. Details of the results are displayed in Figure 1.
d'Arma et al.	2022	VIOLA app modules include news, mental and physical wellbeing, nutrition and educational videos.	Satisfaction	Most participants liked contents of the app and thought it was easy and fairly useful.
Finkelstein & Liu	2018	An interactive telerehabilitation program that supports pwMS adhere to personalized exercise plans at home.	Difficulty, Satisfaction, Evaluation of task operations	Participants reported task difficulty as very low and expressed satisfaction with task presentation, cues, and feedback from the program as high. Most participants successfully completed all tasks in a short period of time.
Finkelstein & Jiazhen	2018	Interactive & personalized telerehabilitation program for pwMS.	Difficulty, Satisfaction, Time to complete.	Participants reported task difficulty as low and expressed satisfaction with task presentation, cues, and feedback from the program as high. Most participants successfully completed all tasks in a short period of time.
Jonsdottir et al.	2018	REHAB@HOME Kinect: A virtual game-based therapy intervention for improving motor functioning and spatial awareness in pwMS.	Feasibility, Satisfaction, Motivation, User experience	Participant’s user experience was positive after each session ranging from 4-4.64/5 for motivation and from 3-4.86/5 for satisfaction.
Midaglia et al.	2019	A smartphone- and smartwatch-based self-administered assessment tool for pwMS, including hand motor function, gait and posture, mood, and cognitive impairment.	Adherence & Satisfaction	Participants showed 70% adherence to active tests and 79% to passive monitoring. Overall participant satisfaction at week 12 was 74.1/100 and remained stable at week 24. Gender was significantly associated to satisfaction.
Palotai et al.	2021	A mobile application for circadian assessment and differentiation of fatigue phenotypes and other mood symptoms.	Adherence & Compliance.	91% of participants completed the questionnaires and 84% completed the questionnaire plus the 14-day VAS and SLD modules.
Tacchino et al.	2015	Cognitive Training Kit (COGNI-TRAcK): A mobile phone and tablet-based application for self-administration of a cognitive rehabilitation intervention based on working memory exercises.	Motivation, Compliance	94% of participants understood the instructions, 100% felt that they could use the app independently at home, 75% found the app interesting, and 81% found the exercises useful. At the end of the program, 81% felt motivated to use the app again clinically.

Thirumalai et al.	2018	Tele-Exercise and Multiple Sclerosis (TEAMS): An at-home tele-exercise program for pwMS comprising of a computer, tablet, and an adjustable floor stand.	Effectiveness, Usefulness, & Satisfaction.	Participants stated that the app was a viable exercise option for individuals with MS to perform at home. Most problems related to the Calendar menu and to visualizations. Minor app issues were noted.
Thomas et al.	2021	A digital homework toolkit for the Fatigue: Applying Cognitive Behavioral and Energy Effectiveness Techniques to Lifestyle (FACETS) program (a 6-session group fatigue management program for pwMS).	Questionnaire and a semi-structured interview collecting feedback about design, content, and functionality.	Good SUS score (74.3), design issues and suggestions for improvement were reported and feedback was subsequently implemented into the DHT.
Tonheim et al.	2018	msHelse: A mobile application for symptoms self-management in pwMS.	Satisfaction, System Usability Scale (SUS), semi-structured interviews with questions regarding the functionalities and the user experience.	High SUS scores, positive group feedback. The mobile diary and summary were reported as the most useful app components.
van Beek et al.	2020	A home-based tablet application for dexterity training for pwMS.	Effectiveness, Satisfaction, & Efficiency	High adherence rate (97%) and absence of dropouts, pwMS scored high on the SUS, were very satisfied with the app features, and found it easy to integrate into their daily lives.
Defer et al.	2018	Vigip-SEP: A mobile application targeting improved reporting of adverse drug reactions in pwMS.	Satisfaction	Not reported.
D’hooge et al.	2018	MS TeleCoach: A mobile telerehabilitation application targeting fatigue by coaching and monitoring physical activity in pwMS.	Satisfaction- D-quest 2.0 Usability Questionnaire	91% of participants completed the questionnaire. Most participants reported being “very satisfied” with a median across most questions of “quite satisfied”.
Pagliari et al.	2021	An at-home virtual-reality rehabilitation system targeting cognitive and motor interventions.	Compliance, Learnability, & Satisfaction	Most participants reported usability in the “excellent range” while the usability and learnability median value were in the 25th–75th percentile range.
D’Aarma et al.	2022	VIOLA: A web-based mobile application for maintaining progress following a multidisciplinary rehabilitation program.	Satisfaction, Usefulness, Likeability, & Ease-of-use	Most participants liked the specific contents, thought the app was easy to use, and found it useful in their everyday life.
Fernández-Vázquez et al.	2021	KSO GT®exoskeleton: A wearable lower limb exoskeleton for gait rehabilitation.	Satisfaction- D-quest 2.0 Usability Questionnaire	PwMS and physiotherapists reported a good degree of satisfaction. Effectiveness, safety, and impact on the patients’ gait were the most highly rated characteristics.
Nasseri et al.	2020	A mobile application providing evidence-based information on the benefits of physical activity for pwCPMS.	Questionnaire assessing patient comprehensibility, general usefulness of the app and it's specific contents.	pwCPMS rated the app as helpful and gave a mean rating of the app’s comprehensiveness of 3.7/5.
Halstead et al.	2020	A telehealth resilience-based skills intervention for pwMS.	Satisfaction questionnaire & engagement.	The mean satisfaction rating of pwMS was 3.65/5 and for support person, 4.76. Qualitative findings revealed technical issues with the program, lack of engagement, the positive aspects of focusing on resilience and cognitive impairment, and a need for the DHT earlier in their MS journey.

Mokhberdezfuli et al.	2021	A mobile application for MS self-management.	Usability questionnaire	The usability and satisfaction were rated as good.
Newland et al.	2016	GAITRite Kinect Gait Sensor: An in-home electronic gait monitoring system.	Feasibility & acceptability interviews	It was shown to be feasible and useful, with some barriers including technical difficulties, privacy concerns, and sensor placements.
Bove et al.	2019	EVOTMis: A digital videogame-based treatment designed to improve attention and inhibitory control.	User experience	Little data reported. Reasons for non-adherence included vertigo (n=1), physical discomfort (n=1) and logical issues (n=1).
Dennett et al.	2020	WEBPaMS: A web-based physiotherapy program.	Qualitative thematic analysis from interviews	3 major themes were reported by participants: 1- convenient & portable, 2 – accessible, 3- flexible expectations
Woelfle et al.	2023	dreaMS: A mobile application & sensors for the remote ongoing assessment of MS symptoms.	Acceptance & meaningfulness	Average daily wear was 91%. Issues with data synchronizations arose for 11% of participants. All tests were reported as meaningful by participants.
Rhodes et al.	2019	MS Performance Test (MPT): A mobile tablet-based assessment program for remote self-assessment of pwMS.	Completion (rate & time, Errors (number & types), & Satisfaction	Completion rate was high (95%) for most modules expect for one. Average completion time was ~33mins & most (85%) were satisfied with their experience of MPT.
Maillart et al.	2020	MSCopilot: A medical software device for the remote self-assessment of pwMS and investigators.	Satisfaction Questionnaire	Most participants (85%) preferred MSCopilot to traditional tests and would use it at home in the future. All investigators would recommend, or use MSCopilot, in clinical settings.
Krause et al.	2022	Levidex: A adaptable digital lifestyle management toolkit for pwMS.	Phone interview & questionnaire assessing practicality, acceptability, & satisfaction.	Participant adherence was good and consistent, and overall satisfaction was rated 74%. Levidex was perceived as highly relevant by pwMS and MS experts.
Stuart et al.	2020	A wearable remote- physiological activity monitoring device for pwMS.	Tolerance (total device wear time) & adverse reactions	The device was well tolerated, wear time was high (average of 99%) and stable over time. Allergic reactions to the metal on the device was reported by two participants.
van Oirschot et al.	2021	MS Sherpa: mobile application & wearable for remote monitoring of walking speed by pwMS and their care providers.	Usability & Learnability interviews	Participants generally reported the program was clear and easy. ~71% of participants experienced some technical difficulties with the application/wearable. Other challenges included emotional fluctuations because of self-monitoring and implementing it into daily routine.
Ouwerkerk et al.	2022	Whereabouts: A remote mobile application to assess pwMS's societal participation.	Effectiveness, Efficiency, & Satisfaction	Whereabouts effectively assessed societal participation (type, frequency, & duration) for 96% of pwMS. 4% of participant activity was inaccurate or incomplete. 57% of pwMS reported being that satisfied with the app with some reporting suggestions for improvement.

Minen et al.	2020	RELAXaHEAD: A mobile app to assess and monitor headaches in pwMS.	Feasibility & Acceptability – recruitment, retention, engagement, & adherence.	There were challenges related to attrition, non-responsiveness, and patient time-demands. A minority of participants found it feasible and acceptable.
van Kessel et al.	2021	MS Energize: A mobile application for self-management of fatigue management in pwMS.	Accessibility & Resonance checking	User experience was reported as positive overall, with feedback provided on the app's design and functioning.
Hsieh et al.	2021	Steady MS: A mobile application for assessing fall risk in pwMS.	Intuitive navigation, Efficiency, & Perceived value	The average SUS score was ~95 across iterations.

PwMS = Persons with MS; pwCPMS: Persons with Chronic Progressive Multiple Sclerosis; SUS = System Usability Scale
